# Geographical Pattern of Testicular Cancer Points to Maternal Exposures Behind Increasing Incidence

**DOI:** 10.1002/ijc.70636

**Published:** 2026-07-04

**Authors:** Antti Kaipia, Karri Seppä, Elli Hirvonen, Eetu Mäkinen, Nea Malila, Janne Pitkäniemi, Jorma Toppari, Peter Boström, Heikki Seikkula

**Affiliations:** ^1^ Department of Urology Tampere University Hospital Tampere Finland; ^2^ Finnish Cancer Registry Institute for Statistical and Epidemiological Cancer Research Helsinki Finland; ^3^ Faculty of Social Sciences Tampere University Tampere Finland; ^4^ Department of Public Health, Faculty of Medicine University of Helsinki Helsinki Finland; ^5^ Research Center for Integrative Physiology and Pharmacology, and Center for Population Health Research, Institute on Biomedicine University of Turku Turku Finland; ^6^ Department of Pediatrics Turku University Hospital Turku Finland; ^7^ Department of Urology Turku University, Turku University Hospital Turku Finland

**Keywords:** cancer epidemiology, endocrine disruptors, environmental factors, testicular cancer, testicular dysgenesis syndrome

## Abstract

Evidence suggests that testicular cancer (TC) originates from germ cell neoplasia in situ arising during fetal development, implying that prenatal environmental exposures may influence disease risk. We investigated whether maternal place of residence during pregnancy is associated with regional variation in TC incidence. We conducted a nationwide cohort study including 1,342,996 men born in Finland between 1960 and 1999. Participants were followed from birth until diagnosis of TC, death, emigration, or the end of follow‐up (2021). A total of 2976 TC cases were identified. Information on municipality of birth was used as a proxy for maternal residence during pregnancy. Incidence rates and cumulative incidence were estimated by birth cohort and age at diagnosis. To examine regional variation, cumulative TC incidence by birth cohort and municipality was modeled using Bayesian hierarchical binomial regression allowing incidence to vary across municipalities. TC incidence increased across birth cohorts, peaking among men born in 1975–1979 (16.8 per 100,000). Cumulative incidence rose in all municipalities, ranging from 67.9 to 132.5 per 100,000 among men born in 1960–1969 and from 283.2 to 327.6 among those born in 1980–1989. Marked geographic clustering was observed. Incidence was consistently elevated among men born in southwestern Finland and remained high throughout the study period. Over successive birth cohorts, an eastward shift in higher incidence was observed. Although direct cohort evidence linking specific environmental exposures to TC remains limited, these findings support the hypothesis that prenatal environmental factors contribute to the pathogenesis of TC and may partly explain regional differences in incidence.

## Abstract (highlights)

What's new?

Testicular cancer (TC) incidence is on the rise in Finland. Though the factors driving its increase remain unclear, prenatal environmental exposures are implicated. Here, the authors investigated TC etiology among Finnish men born between 1960 and 1999 using maternal residence during pregnancy as a proxy for prenatal exposures and applying spatial analyses to cancer registry data. TC incidence increased threefold across birth cohorts and showed persistent geographic clustering, with the highest rates in southwestern Finland and a gradual eastward spread over time. The findings support a fetal origin of TC and suggest environmental exposures may contribute to regional risk differences.

## Graphical abstract

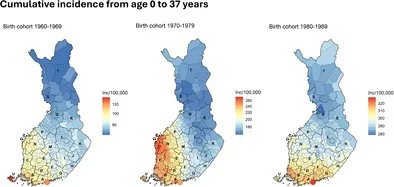

**Abbreviations**
BYM: Besag‐York‐Molie model
CIF: cumulative incidence function
EDC: endocrine disrupting chemicals
FCR: Finnish cancer registry
GCNIS: germ cell neoplasia in situ
GNP: gross national product
INLA: integrated nested Laplace approximation
TC: testicular cancer

## 1 Introduction

The incidence of testicular cancer (TC) has increased in Finland over recent decades, consistent with trends in most Western societies (FCR). Epidemiological data suggest that this rise coincides with increasing gross national product (GNP) [1]. Although the mechanisms linking rising living standards to TC remain unclear, lifestyle changes and exposure to synthetic chemicals are hypothesized contributors [2]. Current evidence indicates that TC develops from carcinoma in situ arising during embryogenesis. Fetal exposure to endocrine‐disrupting chemicals (EDCs) may interfere with germ cell development, leading to gonocyte‐resembling germ cell neoplasia in situ (GCNIS) cells within the testis, which can later manifest as clinical TC [3].

Concurrently, semen quality in Finland has exhibited unique trends. In the early 1990s, marked geographical differences were observed, including within Nordic countries [4]. Denmark reported poor semen quality, whereas Finland showed no decline [5]. Over the past three decades, semen quality in Finland has gradually deteriorated, reducing the gap with Denmark [6]. Notably, this decline initially occurred in southwestern Finland, preceding similar trends in the east [7]. Environmental factors, including EDC exposure, are suspected contributors to declining male fertility [2].

While animal experiments and well‐defined human exposures can demonstrate associations between fetal EDC exposure and gonadal dysgenesis [8], establishing population‐level causality remains challenging [9]. The multitude of chemicals with poorly characterized biological effects complicates causal inference. Geographical analysis can help discern environmental contributions: clustering of cases in a genetically and culturally homogeneous population may indicate local environmental effects. This study examines the fetal origin hypothesis of TC by analyzing maternal residence during pregnancy among TC patients in Finland during a period of rising incidence.

## 2 Materials and Methods

We analyzed TC incidence in a population‐based cohort. TC cases (ICD‐10 C62) diagnosed in Finland between 1960 and 2021 among men born 1960–1999 were obtained from the Finnish Cancer Registry, including birth dates, diagnosis dates, and municipality of birth. Population counts by age, calendar year, and municipality were obtained from Statistics Finland [10].

A total of 2976 TC cases were included. In the analysis of cumulative incidence, 314 cases born abroad or lacking municipality data were excluded. Incidence rates were calculated by birth cohort (5‐year intervals, 1960–1964 to 1995–1999) and age at diagnosis (0–19, 20–29, 30–39, 40–49, 50–57 years). Cumulative incidence was estimated for four birth cohorts (1960–1969 to 1990–1999) by dividing the accumulated cases by the number of men born in Finland. In the 1960–1969 cohort, with complete follow‐up to age 57 for men born 1960–1964, cumulative incidence was estimated at ages 27, 37, 47, and 57. In the 1990–1999 cohort, incidence was estimated up to Age 27.

To examine regional variation, cumulative incidence was stratified by birth cohort and maternal municipality of residence. Analyses were based on the 309 municipalities in Finland in 2021 and on 22 larger districts: 20 hospital districts (with Uusimaa and Helsinki analyzed separately) and the Åland Islands.

Cumulative incidence was modeled using a Bayesian hierarchical binomial regression, allowing variation across municipalities via a BYM model [11] incorporating spatially structured and unstructured random effects. District‐level incidence was estimated as a weighted average of municipality‐specific estimates. Models were fitted separately for the 1960–1969, 1970–1979, and 1980–1989 birth cohorts. Bayesian inference was performed using the R‐INLA package (v24.06.27) applying the integrated nested Laplace approximation (INLA) for posterior estimation [12].

## 3 Results and Discussion

Among the 2976 TC cases, the highest incidence rate (per 100,000) occurred at ages 30–39, with a nearly three‐fold increase from the 1960–1964 cohort (5.7) to the 1975–1979 cohort (16.8), the youngest cohort with complete follow‐up to age 40. Incidence also increased in the 20–29 and 40–49 age groups (Table 1).

**TABLE 1:** Number of men (*N*), number of cancer cases and incidence rate (per 100,000) of testicular cancer in Finland by age and birth cohort.

Birth cohort	*N*		Age group					Total
			0–19	20–29	30–39	40–49	50–59	
1960–1964	201,429	Cases	11	81	111	80	44^a^	327
		Rate	0.3	4.2	5.7	4.2	2.6^a^	2.9
1965‐1969	181,610	Cases	21	106	158	102	13^a^	400
		Rate	0.6	5.8	8.7	5.6	1.6^a^	4.0
1970‐1974	152,842	Cases	22	138	213	116^a^		489
		Rate	0.7	8.9	13.5	8.0^a^		6.4
1975‐1979	165,328	Cases	32	160	294	59^a^		545
		Rate	1.0	9.5	16.8	7.4^a^		7.2
1980‐1984	165,555	Cases	48	232	256^a^			536
		Rate	1.4	13.3	15.3^a^			7.9
1985‐1989	157,606	Cases	35	239	104^a^			378
		Rate	1.1	14.1	12.8^a^			6.6
1990‐1994	166,325	Cases	38	184^a^				222
		Rate	1.1	11.3^a^				4.4
1995‐1999	151,235	Cases	33	46^a^				79
		Rate	1.1	6.3^a^				2.1
Total	1,341,930	Cases	240	1186	1136	357	57	2976
		Rate	0.9	9.3	11.8	6.0	2.3	5.1

^a^ Number and rate are based on incomplete follow‐up data that covered TC cases diagnosed until the end of 2021.

Cumulative incidence rose steadily from the 1960–1969 cohort, peaking in the 1980–1989 cohort. The 1990–1999 cohort exhibits a cumulative incidence similar to the preceding decade, indicating that the TC incidence in Finland has plateaued, a trend seen also in other Western countries (Figure 1) [13]. Regional analysis based on maternal residence revealed marked spatial variation. Regional differences in cumulative incidence became less pronounced as national incidence rose (Table 2). Notably, the increase in incidence was first evident in southwestern Finland. Over time, after peaking in the 1970–1979 cohort, elevated incidence progressively shifted eastward (Figure 2, Table S1).

**FIGURE 1:**
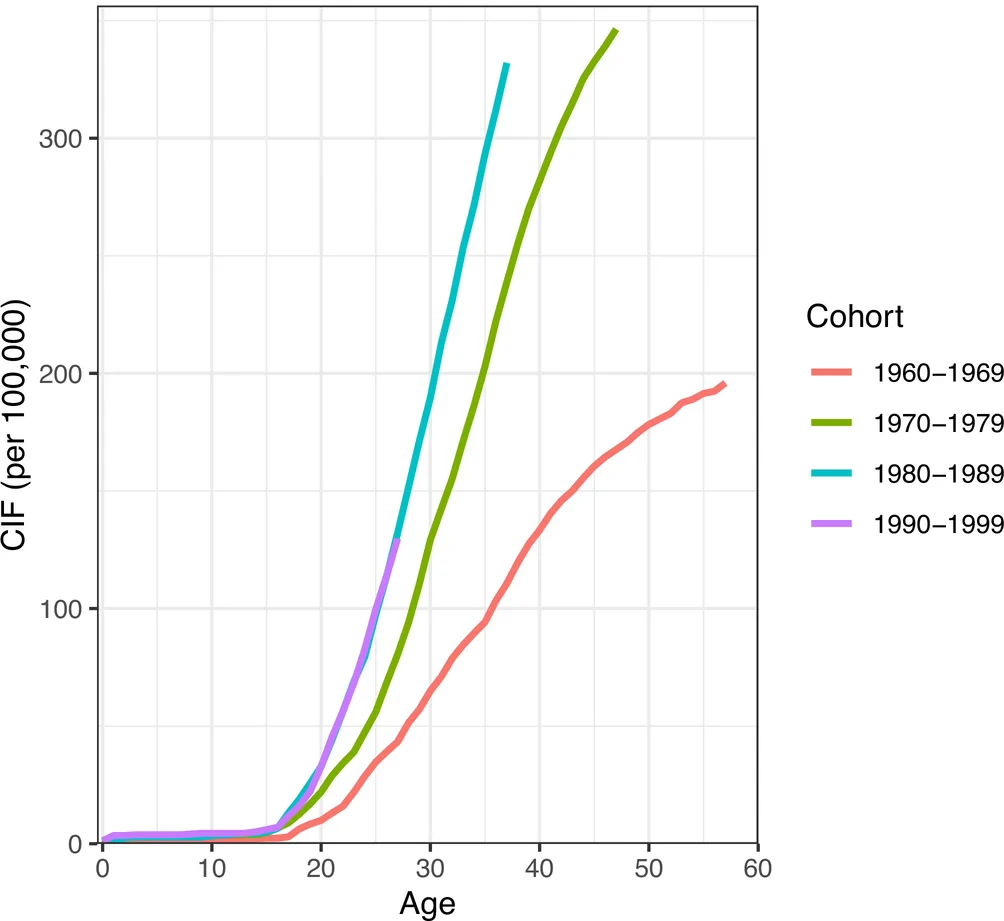
The cumulative incidence of testicular cancer depicted in 10‐year cohorts from 1960 to 1999. CIF, cumulative incidence function.

**TABLE 2:** Cumulative incidence (per 100,000 men; with 95% credible interval) from age 0 to 37, 47, and 57 years, respectively, by birth cohort and collaborative area.

Birth cohort	Southern		Western^a^		Inland		Eastern		Northern	
	Incidence	95% CI	Incidence	95% CI	Incidence	95% CI	Incidence	95% CI	Incidence	95% CI
Age from 0 to 37										
1960–1969	101.1	(85.9, 118.6)	100.4	(82.7, 123.3)	94.1	(80.8, 109.8)	81.0	(65.8, 94.9)	73.2	(53.6, 93.5)
1970–1979	220.1	(197.3, 245.0)	242.8	(210.4, 280.9)	229.1	(203.5, 263.9)	183.8	(155.8, 213.6)	173.8	(141.6, 211.4)
1980–1989	316.4	(289.1, 351.0)	308.2	(281.4, 341.5)	301.6	(276.4, 327.8)	293.1	(260.9, 321.1)	286.3	(240.6, 319.5)
Age from 0 to 47										
1960–1969	166.9	(145.5, 189.8)	161.8	(138.1, 193.1)	149.5	(129.7, 174.0)	115.8	(96.9, 137.4)	101.4	(81.4, 124.5)
1970–1979	321.7	(294.6, 354.9)	334.3	(296.8, 381.5)	316.1	(286.7, 348.5)	279.8	(240.2, 319.2)	275.6	(234.9, 317.7)
Age from 0 to 57										
1960–1969	196.5	(174.2, 220.9)	192.8	(166.2, 223.2)	173.2	(150.7, 198.7)	138.6	(118.6, 159.7)	121	(99.5, 145.6)

^a^ Åland included in Western Finland collaborative area.

**FIGURE 2:**
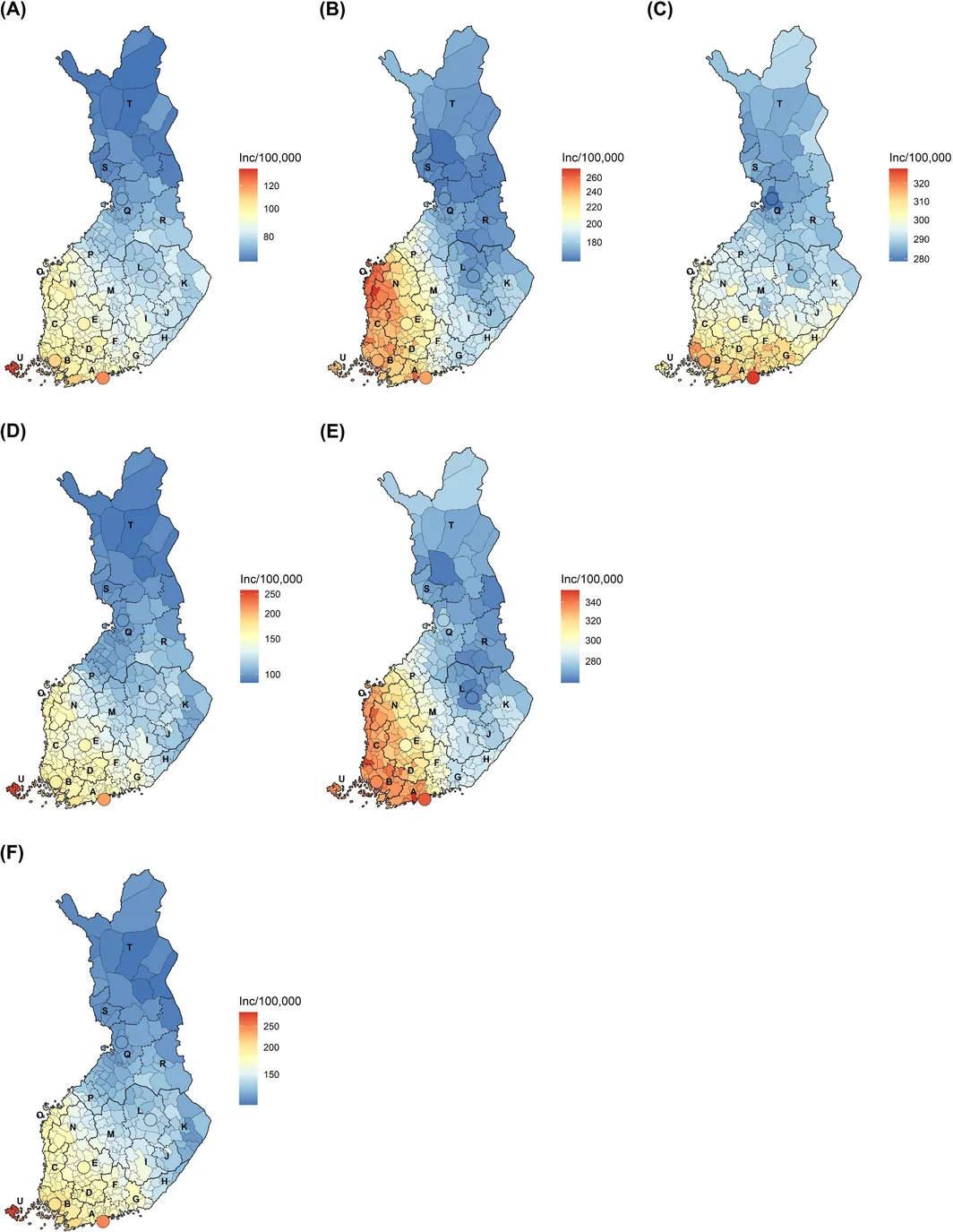
Cumulative incidence of testicular cancer indicated by different colors (incidence/100,000) superimposed on the map of Finland. The alphabets in the map denote hospital regions and they are named in Table S1. (Panels A–C) Cumulative incidence of TC from 0 to 37 years. (A) Birth cohort 1960–1969. (B) Birth cohort 1970–1979. (C) Birth cohort 1980–1989. (Panels D and E) Cumulative incidence of TC from 0 to 47 years. (D) Birth cohort 1960–1969. (E) Birth cohort 1970–1979. (Panel F) Cumulative incidence of TC from 0 to 57 years, birth cohort 1960–1969.

This study demonstrates an approximately three‐fold increase in TC incidence in Finland between men born 1960–1964 and 1980–1984. Analysis of maternal residence during pregnancy indicates that the rise was geographically patterned, first appearing in southwestern Finland before spreading eastward. By the end of the study period, overall incidence plateaued at a higher level, and regional clustering was less pronounced.

Skakkebaek's studies revealed that GCNIS cells, often associated with testicular microcalcifications, resemble fetal gonocytes histologically and immunohistochemically [14]. This supports the concept that GCNIS represents a premalignant condition established in fetal life, which may reactivate at or after puberty to develop into TC [15, 16]. Consequently, TC initiation is considered to occur during fetal gonadal development [17].

Identifying factors predisposing to GCNIS and TC is challenging due to long latency periods and numerous potential environmental exposures with poorly understood reproductive effects. Nevertheless, strong evidence indicates that EDCs can disrupt male gonadal development [17]. The impact of EDCs depends on timing, dose, and chemical properties [18]. EDC exposure is a recognized contributor to testicular dysgenesis syndrome (TDS), which includes hypospadias, low sperm count, infertility, and TC [19].

Although direct causal links between EDC exposure and TDS are difficult to establish, examples such as maternal diethylstilbestrol (DES) exposure illustrate the risks of estrogenic compounds during fetal development. Prenatal DES exposure doubled offspring TC risk and contributed to other TDS manifestations [19, 20, 21]. A wide variety of industrial and agricultural chemicals with endocrine‐disrupting potential are now ubiquitous, likely contributing to observed epidemiological correlations between TC incidence and GNP [1, 18]. Current evidence largely derives from animal studies and cross‐sectional human cohorts, as longitudinal studies linking specific EDC exposures to TDS are scarce. Regional clustering of hypospadias in northern France [22] and elevated testicular tumor incidence in German regions [23] provide indirect support for environmental influences. Previous studies also suggest familial aggregation of TC in Finland, highlighting the potential interaction of genetic susceptibility and environmental factors [24]. The present study's primary finding is the geographical enrichment of TC cases based on maternal residence during pregnancy. Historical declines in Finnish semen quality, first observed in the southwest and now comparable to Denmark, mirror the geographic pattern of TC incidence [7, 25]. These data support the hypothesis that environmental factors contribute to TC risk in utero. Although the evidence remains inconclusive, we hypothesize that the increased incidence of TC in Finland, together with the observed geographical distribution pattern, is primarily driven by environmental factors associated with lifestyle changes accompanying increasing affluence, particularly those characteristic of a “Western lifestyle.”

Strengths of this study include a large, population‐based cohort in a genetically homogeneous population with reliable national registry linkage. Municipality‐level data provide a reasonable approximation of maternal environment during pregnancy, and the Finnish Cancer Registry captures virtually all TC diagnoses, with estimated coverage exceeding 99% [26, 27]. Limitations include the inability to identify specific causal factors underlying the observed spatial patterns, underscoring the need for studies combining spatial epidemiology with direct environmental exposure assessment.

Our findings support the hypothesis that TC originates during critical phases of fetal development, potentially influenced by environmental exposures. Analyses integrating maternal residence during key developmental periods with reproductive health outcomes can provide important insights into environmental contributors to TDS and related conditions, informing future prevention strategies.

## Author Contributions

**Antti Kaipia:** conceptualization, investigation, funding acquisition, writing – original draft, writing – review and editing, supervision, formal analysis. **Karri Seppä:** writing – original draft, methodology, software, data curation, visualization, writing – review and editing, investigation. **Elli Hirvonen:** writing – original draft, methodology, writing – review and editing, software, data curation, investigation. **Eetu Mäkinen:** methodology, data curation, software, writing – original draft, writing – review and editing, investigation. **Nea Malila:** conceptualization, supervision, resources. **Janne Pitkäniemi:** conceptualization, writing – original draft, writing – review and editing, data curation, supervision, resources. **Jorma Toppari:** conceptualization, writing – original draft, writing – review and editing, methodology, supervision. **Peter Boström:** conceptualization, writing – original draft, writing – review and editing, supervision. **Heikki Seikkula:** conceptualization, investigation, writing – original draft, writing – review and editing, supervision.

## Funding

This work was supported by competetive research funding at Tampere University Hospital to the corresponding author (A.K.).

## Ethics Statement

This register study used pseudonymized data obtained from FCR. Permissions to use the data were granted from relevant register holders. According to Finnish legislation, register studies do not require informed consent from participants. All data were handled in accordance with Finnish data protection laws and regulations.

## Conflicts of Interest

The authors declare no conflicts of interest.

## Supporting information


**Table S1:** Cumulative incidence from age 0 to 37, 47, and 57 years, respectively, by birth cohort and hospital district (separately for capital Helsinki and for Uusimaa excl. Helsinki).

## Data Availability

The data that support the findings of this study are available from the corresponding author upon reasonable request.
